# Climate Change and Schools: Environmental Hazards and Resiliency

**DOI:** 10.3390/ijerph14111397

**Published:** 2017-11-16

**Authors:** Perry E. Sheffield, Simone A. M. Uijttewaal, James Stewart, Maida P. Galvez

**Affiliations:** 1 Department of Environmental Medicine and Public Health, Icahn School of Medicine at Mount Sinai, New York, NY 10029, USA; simone.uijttewaal@mssm.edu (S.A.M.U.); james.stewart@mssm.edu (J.S.); maida.galvez@mssm.edu (M.P.G.); 2 Health & Society, Wageningen University, 6708 PB Wageningen, The Netherlands

**Keywords:** school environment, built environment, environmental health, children, students, health effects of climate change, vulnerability, adaptation, mitigation, disaster preparedness

## Abstract

The changing climate is creating additional challenges in maintaining a healthy school environment in the United States (U.S.) where over 50 million people, mostly children, spend approximately a third of their waking hours. Chronic low prioritization of funds and resources to support environmental health in schools and lack of clear regulatory oversight in the U.S. undergird the new risks from climate change. We illustrate the extent of risk and the variation in vulnerability by geographic region, in the context of sparse systematically collected and comparable data particularly about school infrastructure. Additionally, we frame different resilience building initiatives, focusing on interventions that target root causes, or social determinants of health. Disaster response and recovery are also framed as resilience building efforts. Examples from U.S. Federal Region 2 (New Jersey, New York, Puerto Rico, and the U.S. Virgin Islands) and nationally are used to illustrate these concepts. We conclude that better surveillance, more research, and increased federal and state oversight of environmental factors in schools (specific to climate risks) is necessary, as exposures result in short- and long term negative health effects and climate change risks will increase over time.

## 1. Introduction

In the United States, 50 million children join 3.1 million teachers to attend school for an average of 30 h a week, or approximately a third of their waking hours [1]. Time in school has increased over the past twenty years, becoming, outside of the home, the most influential environment on a child’s health and wellness [2,3]. Despite this, amidst all the competing interests within the school setting, environmental protections have rarely taken precedence [4]. Now, with a changing climate, additional challenges arise in maintaining a healthy school environment [5]. Climate change is the changing of weather patterns due primarily to the increase in carbon pollution in the atmosphere. These altered weather patterns include rising temperatures, heavy rains and droughts, and some other kinds of severe weather [6]. Globally, children are the most vulnerable to climate change, bearing 88% of the burden of disease resulting from climate change [7].

The school built environment, comprising building infrastructure, grounds, neighborhood and surroundings—not just the instruction, relationships, and other significant experiences that occur in school—is critically important and can promote health or introduce harmful exposures that significantly impact children’s well-being [8]. Commonly encountered environmental hazards are indoor exposures that include, but are not limited to, chemical exposures such as lead, mercury and polychlorinated biphenyls (PCBs) from building materials or items intentionally brought into the school for maintenance or teaching (i.e., science curriculum), indoor air pollution, exposures to mold, exposures to noise, and drinking water contamination [9]; and outdoor exposures that include, but are not limited to, outdoor air pollution and contaminated soil (influenced by geology and past land use near school location), and physical and other nearby hazards [4]. Climate change can make existing problems in schools worse (e.g., worsen indoor air quality due to mold growth or increase risk of exposure to toxic building materials post flooding), and increasing evidence suggests potential for climate change to also introduce nascent environmental hazards (e.g., heat extremes in previously cooler climates, overcrowding due to displaced populations, or shifting geographic range of vector borne diseases) [10].

Children today are affected by the ‘new pediatric morbidity’ a term that heralds immense success in regards to fighting infectious diseases and also new challenges such as the now common conditions in childhood: asthma, attention deficit hyperactivity disorder, and obesity among others [11]. Children’s respiratory health, neurocognitive development, immune system response, their learning comprehension and school performance, and even adult health status and life expectancy are all influenced by their school environment [9,12,13]. Certain children are more vulnerable than others. For example, poor indoor or outdoor air quality can cause or worsen respiratory illnesses, such as asthma, and is associated with headaches, dizziness, tiredness, and difficulty concentrating which can compound learning challenges for students with underlying neurocognitive disorders. Environmental exposures and sickness lead to missed schools days and work days for their care givers [14].

Extreme precipitation, heat waves, sea level rise, changing geographic and seasonal patterns of plants, animals (particularly insects), and disease-causing microbes, and food insecurity (including changing nutritional content) impose health hazards that compound day-to-day school exposures [15]. As evidenced by recent super storms Harvey, Irma and Maria, new environmental problems arise when functional school infrastructure is compromised and previously well contained hazardous materials are spread by floods. Such disasters, made more frequent and more extreme by climate change [16], increase mold growth in classrooms, contaminate school grounds, create physical and chemical hazards from debris and downed electric wires and toxic substances, and result in prolonged school closures with pervasive social and economic impacts for families and communities.

Additionally, historical and on-going structural racism—“the totality of ways in which societies foster racial discrimination through mutually reinforcing systems of housing, education, employment, earnings, benefits, credit, media, health care, and criminal justice” [17]—has pervasive effects as well. The far reaching effects of structural racism include contribution to health and educational disparities and the vulnerability of the students in schools with significant built environment concerns, resource limitations that lead to poor maintenance, infrastructure deterioration, and toxic environmental exposures. Children of poverty and communities of color bear a disproportionate burden of environmental hazards [18] and often attend schools with the most strained resources, have a higher risk of lower school readiness and impaired school performance, and then higher punitive discipline and greater criminal justice system involvement and subsequent reduced graduation rates. Climate change creates additional stressors through disruptions to the basic educational process that compounds this cycle of poverty and health disparities [19,20,21].

In this paper, we illustrate climate change related risks to the infrastructure of schools and consequently children’s health. Secondly, we explore current school environmental hazards prevention and planning efforts in the context of a healthy school movement. As available data allows, we use examples from Federal Region 2: New Jersey (NJ), New York (NY), Puerto Rico (PR), and the U.S. Virgin Islands (USVI) to illustrate the heterogeneity across a single administrative region.

## 2. Methods

We focused on specific vulnerabilities associated with climate change and the school built environment and paid particular attention to specific geographic regions, and resilience efforts related to schools. Databases included Pubmed and Scopus to capture literature from the biomedical, environmental health, and educational fields. In addition to specific search engines, our search also included a snowballing strategy using the references from some of the cornerstone reports and peer-reviewed literature relevant to our cross-disciplinary topic. We referenced empirical studies, review articles, commentaries, news articles, and organizational reports to obtain information on schools and student populations. We included search terms that encompassed climate change, global warming, and resulting extreme weather such as flooding. We used the terms “school infrastructure OR school built environment” to find references relevant to the permanent school buildings, temporary structures such as mobile trailers, playgrounds, other facilities essential for school functioning or where students spend time such as athletic fields, and routes to and from school.

School statistics were obtained by using most recent datasets from National Center for Education Statistics (NCES). However, as most national datasets do not include data on Puerto Rico and the USVI, we used grey literature, governmental websites, Google, and Google Scholar to cast a broader search net. Search terms included phrases such as “school maintenance OR school safety hazards OR climate resilience AND Puerto Rico OR US Virgin Islands” as well as “school resilience,” “building back better,” and “schools after hurricanes.” We further focused on the climate related risks of extreme weather and sea level rise that have specific impact on school infrastructure, noting that there are many climate change associated environmental implications, such as shifting insect habitat. An additional climate change risk is that of extreme heat which is of particular concern for student athletes [22] and at schools in warming climates without air conditioning [23]. Given that the risks of extreme heat are well documented [24], we chose to highlight less addressed climate change risks, and, thus, mention the risk of increased temperatures only minimally in this manuscript.

## 3. Results

### 3.1. Climate Change Risks

**Figure 1:**
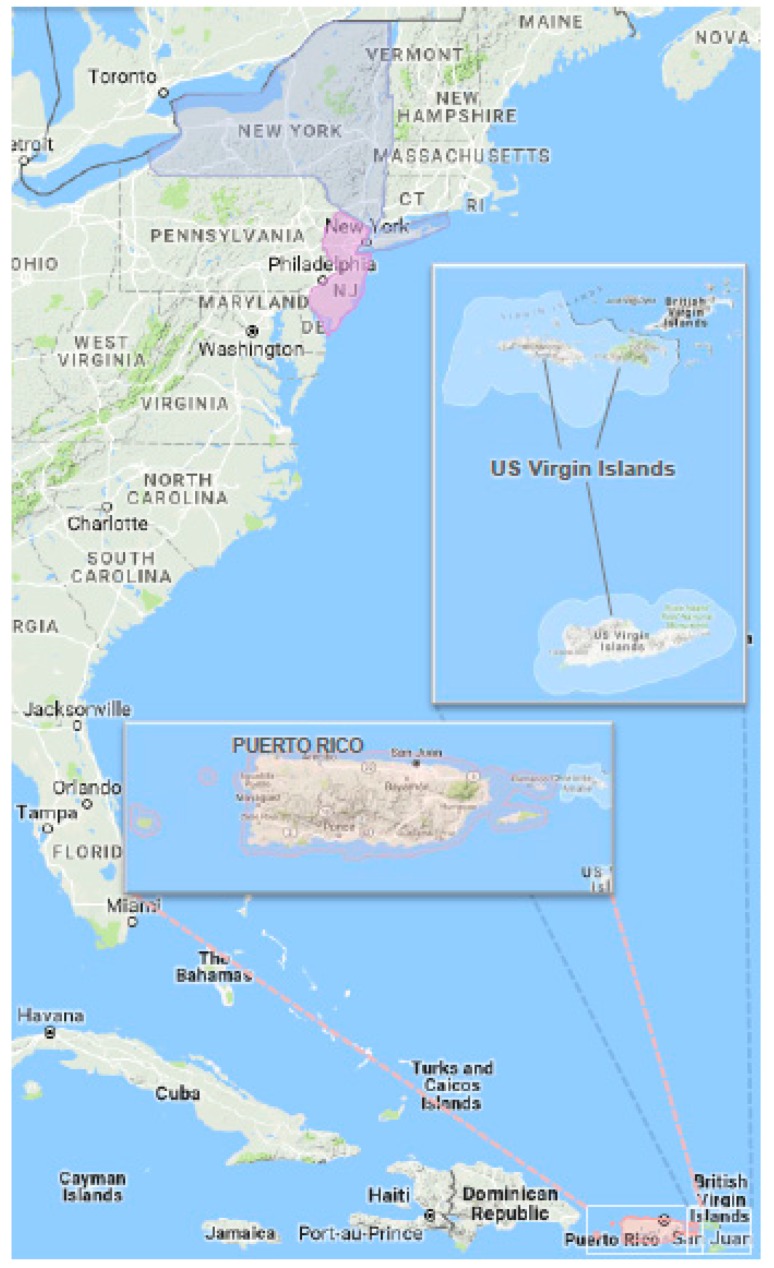
Map of U.S. Federal Region 2: New Jersey (NJ), New York, Puerto Rico, and U.S. Virgin Islands (image created from Google Maps).

According to the World Health Organization, children, people living in megacities, and people living on islands, are most at risk of health effects from a host of climate-related changes in extreme weather. Children going to school in PR, USVI or NYC and other megacities are particularly vulnerable [25,26]. While climate change impacts will differ across Federal Region 2 (Figure 1) due to differences in baseline climate, resources, demographics, population density, local ecology, and geographic terrain, climate change in general will bring greater risk of heavier storm events and resultant flooding and property damage separated by hotter and drier periods [27]. New York and New Jersey could see significantly shortened winters by the end of the century with a shift toward rain instead of snow in previously snowy regions. Most climate models also predict longer periods of extreme heat—a condition that is further intensified by the urban heat island effect [28]. Puerto Rico and the USVI share heat, flooding, and sea level rise risks with parts of New York and New Jersey but also have elevated risks from tropical storms and hurricanes [29], and specific vulnerabilities to their fresh water supply, diminished resources, and limited mobility because of being small islands [30] further complicated by their non-statehood status [29]. Because of their dense urban coastal populations, New York and New Jersey have the majority (63%) of the people at risk from coastal storms and sea level rise in the Northeastern U.S. [31].

Climate change risks such as extreme precipitation and sea level rise are also problematic for contaminated or toxic material containing areas. EPA identified superfund sites involving groundwater remediation and on-site systems for contaminant source containment located within 100-year and 500-year floodplains as areas of particular risk of disturbance from climate change [32]. Within these locations, vulnerability to high winds, storm surge, flooding, heat, and chemical hazards is not distributed evenly, as built environment, condition of human settlements, and baseline health are determinants of socioeconomic vulnerability [33].

### 3.2. Current State of Schools and Students: Case Study of Federal Region 2 and National Examples

#### 3.2.1. Failing School Infrastructure

U.S. Federal Region 2 (Figure 1) consists of two Northeastern states (New Jersey and New York) located in a temperate climate and two island entities, Puerto Rico (a commonwealth) and USVI (a territory) in the Caribbean tropics. Table 1, shows specific Federal Region 2 area numbers for schools and students (discussed more in Section 3.2.2) with national numbers provided for context. The Region has about 5% of the total U.S. public elementary school student population [34,35,36,37]. The information presented compares only at the state, territory, and commonwealth area though we acknowledge that intra-entity variation (i.e., within each of these entities) might be as much or greater than inter-entity variation.

We identified only three national level studies that assessed school building infrastructure in the last three decades, none of which provided data for U.S. entities outside of the 50 states. In a 1995 U.S. General Accounting Office study, more than half of U.S. K-12 public schools reported at least one building feature needing extensive repair with HVAC and plumbing being the most frequently reported; and almost two thirds reported at least one unsatisfactory environmental factor with acoustics for noise control, ventilation, and physical security being the most reported factors [48]. A 2014 study by the U.S. Department of Education showed that infrastructure problems still affect the majority of school buildings and their outdoor facilities. These findings for the Northeast region were consistent with the national averages. The same study indicated that nationally 99% of K-12 public schools and 100% in the Northeast have at least some permanent buildings of which the average building age is 44 years, and 31% of schools nationally while only 12% within the Northeast have portable buildings [51] which present different management challenges. Most recently, a 2016 report by the non-profit 21st Century School Fund found an annual underinvestment of $46 billion to assure a healthy and safe environment in K-12 public schools. Over the period 1994–2013, New Jersey and New York ranked in the top three of U.S. states on spending for maintenance and operation of school facilities measured per student ($1923 and $1759 respectively) and per square foot. These figures included the combined costs for utilities, routine maintenance, cleaning, minor repairs, and security [52].

Overall, the Caribbean entities of Federal Region 2 had scant data from mostly anecdotal sources. Over a decade ago, Puerto Rico was cited for violations related to federal asbestos regulations in school buildings [53]. A Health Impact Assessment in an environmental justice community of San Juan documented frequent, recurrent, and disruptive flooding affecting school attendance and school building infrastructure [54]. In 2013, the Caribbean Environmental Protection Division undertook an assessment of Puerto Rico’s schools to examine the prevalence of common health hazards. Initial findings report only that peeling ceiling plaster has been found in numerous classrooms without additional details [55]. A letter indicating near completion of the study was sent to the Puerto Rican Department of Education in 2016, though a final report was never issued because of lack of funding [56]. The current financial crisis in Puerto Rico has led to the closing of dozens of schools and further challenges to maintenance of school infrastructure. The Puerto Rican Secretary of Education reported decrepit facilities and rat infestations, and the Puerto Rican teachers’ union president said, “Everyone recognizes that some schools are in deplorable conditions…” [57]. The subsequent 2017 hurricane season, specifically Hurricanes Irma and Maria, wreaked havoc across the island, shuttered many schools for weeks or longer, and highlighted the resource the physical structure of schools can play as shelters or community gathering places in the aftermath of storms [58,59].

Regarding the USVI, a 2013 assessment of K12 public school buildings and classrooms for US Insular Areas (Commonwealth of the Northern Mariana Islands, Guam, American Samoa, and USVI) reported that the total costs of deferred maintenance in K-12 public schools on the Virgin Islands are about $66.2 million and that the average age of school buildings was 40 years (consistent with the national average noted above) [60]. The report assigned facility scores to the public schools on a scale from 1 to 5; the USVI had an overall score of 3.6, comparable to the other areas assessed. Key problems in the USVI include corroding rebar, spalled concrete, deteriorated wood elements, weatherproofing, air quality concerns, plumbing leaks, exposed electrical elements, vehicle circulation, emergency vehicle access, fire protections (including fire hydrant provision), and site drainage. Facility items that should be standard but were missing from at least some of the school buildings in the USVI include: PA system, fire alarm, fire hydrants, backflow preventer, emergency vehicle access, fences, and gates. Additional site concerns consisted of site drainage and flooding problems and poor condition of asphalt and pavements.

#### 3.2.2. Underlying Vulnerability: School Location and Student Health

School location in relation to the surrounding properties’ current and historical use is an important consideration as well. Outdoor air, water, and soil quality can be influenced by Superfund sites, solid waste landfills and transfer stations, active industrial sites, and other chemical storage sites located proximal to schools [61]. Young children and pregnant women, due to rapid and sensitive development in early life, are most at risk from toxic chemical exposures [62]. Many schools are located adjacent to toxic sites that represent potential hazardous exposures, particularly in extreme weather conditions. Notably in the U.S., there are over 12,000 so called “fenceline” schools that are within one mile of high-risk industrial facilities using or storing large quantities of toxic chemicals (i.e., those involved in the Risk Management Program). These schools serve nearly 1 in 10 school children. In New York, 2.8% of the population is living in one of the fenceline communities, a relatively small percentage compared to other states in the US. In New Jersey, 4% of the total population is living in a fenceline community. Poor, Black children (age < 12) are 2.5 times more likely (compared to white children not in poverty) to live in a fenceline community and Black children overall are 1.4 times more likely to attend a public school in a fenceline community. In New York, poor Black children are 1.7 times more likely to live in a fenceline community [63].

Another key vulnerability factor is poverty and the underlying health status of children which affects their susceptibility to environmental exposures. Poverty rates—represented by children eligible for free lunch—vary highly across the different geographic regions with Puerto Rico having the highest rate at >90% of children [36]. Some of the disease epidemics affecting children today in the U.S. such as asthma and prematurity occur above national rates in Federal Region 2 (Table 1). And, furthermore, high prevalence of underlying student illnesses and vulnerable school location can compound each other when disasters occur. When Hurricane Floyd struck the eastern U.S. coast in 1999, low income schools serving a majority of Black students had twice the risk of being flooded compared to majority non-Black, non-low income schools. The authors of that study posited that the root causes of this increased risk dated back to post-Civil War when Black families settled in the less desirable, often lower lying and flood-prone land and that such inequality was perpetuated by subsequent racial segregation laws. Flooding and resulting mold risk posed a particular risk to the students in these schools given the high rates of respiratory illness [64].

### 3.3. Climate Change Resilience in Schools

Resilience is defined as “the ability of a social or ecological system to absorb disturbances while retaining the same basic structure and ways of functioning, the capacity for self-organization, and the capacity to adapt to stress and change” [65]. Thus, climate resilient schools, even in the face of significant disturbances such as severe weather, remain places where caregivers entrust the care of their children for the purpose of learning. This section presents climate change-related preparedness and response efforts that increase resilience at U.S. schools, again using Federal Region 2 examples where possible but drawing more national examples for breadth [66].

#### 3.3.1. It Takes Roots to Weather the Storm

**Figure 2:**
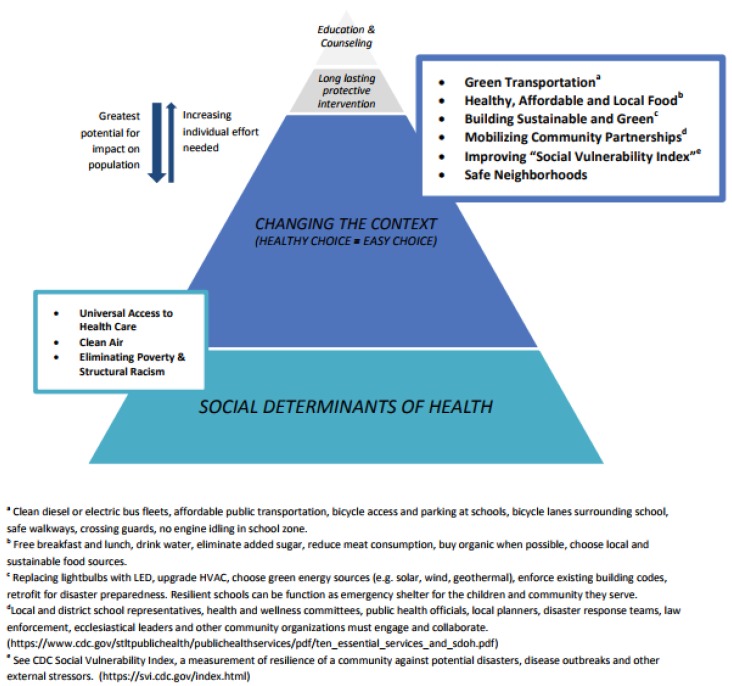
Health Impact Pyramid with climate change preparedness efforts that influence schools or the communities in which schools exist (modified from Frieden [80]).

Preparedness refers to actions being carried out to build the capacity to manage a disaster and ensure effective response [67] or even minimize the response needed. Preparedness activities are forms of prevention and take place on multiple levels as illustrated by the health impact pyramid shown in Figure 2. Activities that target an individual (represented by the top of the pyramid) take considerable effort while affecting the smallest portion of the population. Conversely, efforts targeting social determinants of health (the base of the pyramid) have greater implications for a population. If the analogy is a tree instead of a pyramid, those actions that affect the social determinants of health are strengthening the roots.

At the individual school level, preparedness for disasters occurs in different ways. As occurred in Puerto Rico post-Hurricane Maria, many times the sturdy construction of schools means they can serve as shelters or community centers post-disasters. This reality can be by accident or design. In the state of Kansas, for example, multipurpose tornado shelters, with cafeteria, gymnasium, library and other facilities, share space with local schools. Construction of the shelters was supported by a Federal Emergency Management Agency (FEMA) grant, covering 75% of the costs and remaining 25% from non-governmental sources. For this project in Kansas, including a shelter in the building process of a new school increased the total budget 2.5–3.5%. One shelter housed in an elementary school was used three times during its first three months of operation [68].

To improve the scalability of such individual school efforts, some organizations have collected successful case studies and developed guidelines on resilient infrastructure. The “Guidance Notes on Safer School Construction” by the Global Facility for Disaster Reduction and Recovery (GFDRR) provides information about the needs and steps for establishing safer schools and basic design principles for different disasters (e.g., earthquakes, windstorms, floods, landslides and wildfires) [69]. Additionally, the EPA “School Siting Guidelines” help evaluate school siting locations and address renovation concerns, and can be used by parents, teachers and communities to call attention to large industrial facilities, distribution centers, bus terminals, truck stops, high-traffic roads and highways, and former defense sites as important considerations during school site selection [61]. A third resource, the “US Climate Resilience Toolkit” presents domestic case-studies including four studies from New York, two from New Jersey, and 46 nationally related to the built environment [70].

Interventions targeting the social determinants of health (the base of the pyramid) have the biggest potential impact on health whether or not they specifically address school infrastructure. As one U.S. Federal Region 2 example, New York City is a member of the “100 Resilient Cities” program, an initiative by the Rockefeller Foundation, and has released its resilience strategy, including how to deal with a growing population, improve social and economic equity, and increase sustainability. The resulting investments of this strategy also include strengthening school infrastructure at some locations but not city-wide [71]. In Puerto Rico, the complex disaster of a decade long recession, the more recent debt crisis, and Zika virus epidemic [72], is now further compounded by massive infrastructure damage by Hurricane Maria, and federal policy response will likely be the largest driver of poverty levels across the island in subsequent years as it has been in the years leading up to the current fiscal crisis [73].

In U.S. Federal Region 2 and beyond, current initiatives suggest reforming the financial structure of preparedness and response to better address these root causes. “Re-focus”, a group of social-entrepreneurs, suggests a new link between catastrophe bonds and insurance coverage which results in “resilience bonds”. Resilience bonds can combine the two traditional investments in order to bridge the gap between preparedness and response. This market based model, while unproven, has potential to support proactive investments in prevention and generation of resilience dividends [74]. Other financing models include Pay for Success (PFS) where private investors pay upfront for a social service that is then repaid if pre-specified health, economic, or social outcomes are met. Groups such as Green and Healthy Homes Initiative already lead numerous asthma-focused PFS projects [75]. The PFS model could be expanded to include climate-resilient health initiatives more broadly. More traditional funding mechanisms such as grants for financing healthy school environments are compiled for reference by the EPA [76]. Importantly, preparedness efforts may also result in prevention efforts on climate change and vice versa and, consequently, may have co-benefits for public health [77,78]. Furthermore, secondary benefits of preparedness can also lower school operations costs for energy and water and improve student health and teacher retention [79]. Thus, financing toward any of these efforts might have concomitant benefits in other ways.

#### 3.3.2. Building Back Better

**Figure 3:**
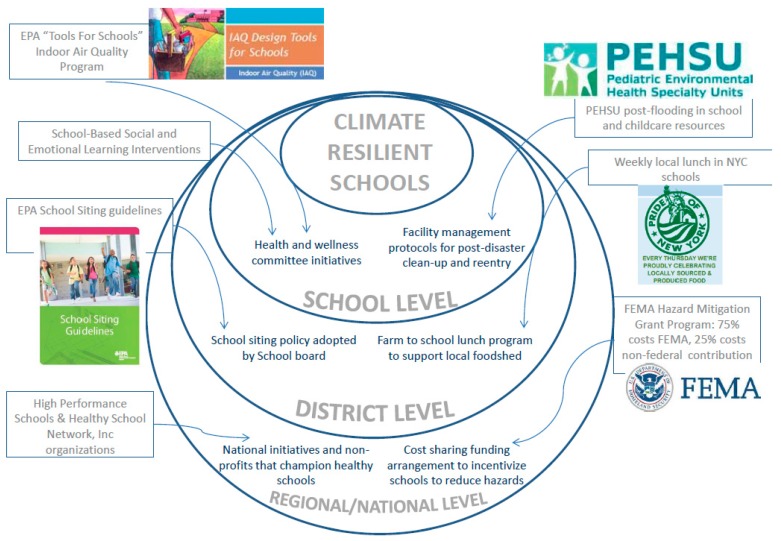
Socio-ecologic model showing the multiple levels from individual school to regional/national initiatives that influence schools’ resilience to climate. Adapted from Ecological Systems Theory model [78].

Extreme weather events are projected to worsen in the coming decades [65]. Such events, regardless of the level of school preparedness and climate change mitigation, will continue to test the resilience of school infrastructure and their supporting systems. For USVI and Puerto Rico in particular, extreme weather is recurrent and even, in some cases routine, as with the annual hurricane season from June through November in the Atlantic and Caribbean. The response and recovery phases after devastating disasters determines much about how a school and the surrounding community will fare during the next event [81]. Building back better—learning from each event and designing vulnerability out of the system—is paramount. There are multiple levels on which response and recovery activities occur as shown in the socio-ecologic model in Figure 3.

At the national level, two key guidance documents create a road map for recovery that includes an emphasis on health: the National Disaster Recovery Framework (NDRF) and the National Academies report on Communities after Disasters. The NDRF is “a guide that enables effective recovery support to disaster-impacted States, Tribes, Territorial and local jurisdictions” [82]; and the National Academies report emphasizes the importance of incorporating school systems into all levels of preparedness, response, and recovery planning and activities [81]. Also at the national level, FEMA provides grants that fund recovery activities beyond the immediate disaster response period and a design guidance document that complements community level efforts to restore school function post-disasters and improve the baseline vulnerable functionality [83,84]. Furthermore, there are national networks, non-profits, and other civil sector organizations, such as the Pediatric Environmental Health Specialty Units (PEHSU), US EPA Office of Children’s Health Protection, Centers for Disease Control and Prevention/National Center for Environmental Health, Collaborative for High Performance Schools, and Healthy Schools Network, Inc., that have considerable expertise and are poised to serve in an advisory capacity regarding child health and safety in schools after disasters [4,85]. Also, at the national level, disasters can serve as the impetus to review and change policies that influence underlying vulnerability such as The National Flood Insurance Program (NFIP); state insurance programs and private sector risk sharing [86].

At the community or school district level, more tailored geographic responses help build resilience. For example, “Rebuild by Design” is an organization resulting from a design competition held after Superstorm Sandy, launched by the US Department of Housing and Urban Development (HUD) in partnership with the philanthropic sector and nonprofits. It brought physical, social and community experts together for research and design. Examples of their work are “Climate Ready Boston” and a current project in San Juan, Puerto Rico [87,88]. Furthermore, for municipalities to take advantage of previous successes in infrastructure improvements, the on-line resource called “The Atlas” gives an overview of more than 250 projects and research—some of which are school specific such as a school district’s implementation of LED lighting for energy savings and lighting quality benefit. These projects aim to create stronger, safer, and more sustainable infrastructure [89]. The NYC Blue & Green Roof Pilot project, for example, compares vegetated and non-vegetated roofs storm water runoff. The “green” and “blue” roofs both have capacity to store rainwater to prevent overflow of sewers and recreational water contamination during storms [90].

Also at the district and municipality level, disasters can serve as an opportunity to address underlying inequities. For example, after Hurricane Katrina, school policies in New Orleans changed radically with the intention of lifting New Orleans schools out of crisis. Before Katrina, school governance changes had already been proposed to resolve the crisis, but Katrina ‘kicked them into overdrive’ [91] increasing the proportion of non-profit charter schools in the city. While studies disagree to the extent that such changes overcame educational disparities and outcomes, the history highlights disaster as an opportunity for change [92,93] and further underscores that effective long-term recovery requires an emphasis on resilience, overcoming structural inequalities, and long term commitments of funding and other support, not only short term reconstruction [79].

At the individual school level, informational resources such as those available from the PEHSU network on children’s health in the aftermath of floods [94] and returning to impacted areas [95] can help guide school decision makers and committees. The EPA Indoor Air Quality Design Tools for Schools offers help to school districts and facility planners to improve the indoor learning environment [96]. And, in terms of psychosocial and emotional resilience, multiple institutes and initiatives, such as Collaborative for Academic, Social, and Emotional Learning (CASEL), University of Southern California National Center for School Crisis and Bereavement, and University of California Santa Barbara’s Mental Health Matters curriculum, are supporting school-based social and emotional learning that can bolster school communities before, during, and after disasters [97,98,99].

## 4. Discussion

We found that climate change worsens existing environmental health issues in schools, presents new challenges and also affords opportunities to implement health promoting opportunities while increasing resilience. The potential health impacts include worsened childhood asthma, cognitive and learning issues, mental health consequences, and missed school days for children and work days for caregivers. The risks of such impacts vary by geographic area based on projected weather and ecological changes secondary to climate change; they vary by school district and individual facility based on baseline condition of the school built environment; the risks vary based on which hazards occur in proximity to the school; and, finally, they vary based on existing resource disparities across communities resulting from on-going structural inequalities and chronic low prioritization of these issues. The examples of emerging resilience underscore our finding that, with careful planning, disasters themselves can serve as opportunities to become more resilient to the risks posed by climate change and to improve overall student well-being and school performance. Specifically, we observed that rates of underlying childhood disease and risk factors and condition of school infrastructure vary both within and between jurisdictions, highlighting the need for local vulnerability to environmental exposures to also be used in prioritizing adaptations.

Our investigation also revealed that quality and availability of data on health, environmental risks, and school infrastructure vary significantly by political geography. No systematic indicators for accessing environmental health in schools exist in the U.S. which prevents direct comparisons of indicators between regions. Such indicators could be used for both baseline assessment as well as tracking progress of interventions or impact of policies. Furthermore, we also acknowledge that disparities within a single geographic area could be as much or more than between areas but that investigation was outside the scope of this project. This paper illustrates a starting point and the current landscape on which the need for future research and advocacy can be based.

In Region 2, and in the U.S., different organizations offer guidelines for protecting children’s health in schools, but such guidelines and programs are voluntary and not mandated. Our findings further support the recommendations made by Barnett and Paulson [4] and the American Academy of Pediatrics [100], stating a federal/national environmental public health agency should have the authority and budget to establish a program addressing children’s environmental health, covering school environmental health, which allows for research of climate-associated health effects, supports education and awareness of school environmental health issues, promotes sustainable energy use, and supports development of local adaptation strategies. The health sector, representatives across federal agencies (for example, US EPA, CDC/NCEH, NIEHS, FEMA, DHHS, HUD), parents and mental health advocates should all be included in this agency. Such a strategy can have co-benefits on health in the short- and long term.

While we focused on Federal Region 2 for illustrative purposes, even more differences arise, in exposure to climate change, disparities, existing resilience efforts, maintenance of schools, and ultimately child health when considering the U.S. national level [101]. Such disparities are even greater on a global level [13]. Inhabitants of islands and people in developing countries are most at risk. In developing countries, school infrastructure is less resilient to climate disasters, putting children even more at risk [30], and furthermore, there are important resilience examples from which the U.S could draw [102]. In addressing the gaps in terms of climate resilient schools, the U.S. therefore has an opportunity to learn from, model, and support such an effort globally.

## 5. Conclusions

We studied the risks that climate change poses to children in schools in U.S. Federal Region 2 and the potential that resilience efforts present to improving overall student and community well-being. The disparities seen at the level of Federal Region 2 are manifest nationally and even more so globally. We emphasize that “It takes roots to weather the storm” by focusing on opportunities to change the context and affect social determinants of health; and we frame response and recovery initiatives by focusing on the concept of resilience through “building back better”. In order to prevent further structural racism and socioeconomic disadvantages, special attention should be on areas prone to climate change related disasters, and on disadvantaged schools and children, as they are more vulnerable to climate change risks. Better surveillance, more research, and federal oversight of environmental factors in schools (specific to climate risks) is necessary, as exposures result in short- and long term negative health effects and climate change risks will increase over time.

## Figures and Tables

**Table 1:** U.S. Federal Region 2 area numbers for students and schools.

Topic	National	New Jersey	New York	Puerto Rico	U.S. Virgin Islands
Public school students (elementary and secondary)	50.3 million ^a^	1,400,579 ^a^	2,741,185 ^a^	410,950 ^a^	14,241 ^a^
Public elementary school students	35.3 million ^b^	495,307 ^c^	1,010,166 ^c^	269,419 ^d^	5352 ^g^
Students eligible for free/reduced-price lunch (%) *	51.3 ^f^	35.5 ^f^	47.9 ^f^	91.4 ^e^	>39.3 ^¥^
Children with Asthma (lifetime diagnosis) (%)	4.7 in 0–4 yo 9.8 in 5–14 yo ^h^	8.4 in 0–4 yo 13.4 in 5–9 yo 18.1 in 10–14 yo ^h^	7.8 in 0–4 yo 19.5 in 5–9 yo 18.7 in 10–14 yo ^h^	17.0 in 0–4 yo 30.4 in 5–9 yo 22.5 in 10–14 yo ^h^	No data
Uninsured children (%)	5 ^i^	3.9 ^i^	2.6 ^i^	3.4 ^l^	19.2 ^m^
Children with ADHD (currently diagnosed) (%)	8.8 ^j^	5.5 ^j^	7.7 ^j^	7.5 ^k^	No data
Prematurity (%)	9.63 ^n^	9.80 ^n^	8.70 ^n^	11.4 ^o^	12.7 ^p^
Public schools (elementary and secondary)	98,373 ^a^	2571 ^a^	4826 ^a^	1378 ^a^	30 ^a^
Schools with ≥1 inadequate building feature (%)	57 ^q^	53 ^q^	67 ^q^	No data	No data

^a^: 2014–2015 [1]; ^b^: 2013 [34]; ^c^: Calculated for grades 1 to 5, 2014–2015 [35]; ^d^: 2009–2010 [36]; ^e^: K12, 2009–2010 [36]; ^f^: PK12, 2012–2013 [38]; ^g^: 2015–2016 [37]; ^h^: 2013 [39]; ^i^: age <19, 2015 [40]; ^j^: age 4–17, 2011 [41]; ^k^: age 4–17, 12-month prevalence rate, 2007 [42]; ^l^: age <18, 2014 [43]; ^m^: age 6–17, 2009 [44]; ^n^: 2015 [45]; ^o^: 2016 [46]; ^p^: 2012 [47]; ^q^: 1995 [48]; * For the 2013 school year a child in a household of four persons would be eligible for free lunch if the family’s annual income is no more than $29,965, and for reduced-price lunch if the family’s income does not exceed $42,643 [49]. ^¥^ Calculated from Census results, 39.3% of families have an income <$34.999, 27.4% of the families have income <$24.999 [50].
